# Primary care physician volume and quality of care for older adults with dementia: a retrospective cohort study

**DOI:** 10.1186/s12875-021-01398-9

**Published:** 2021-03-09

**Authors:** Natasha E. Lane, Vicki Ling, Richard H. Glazier, Thérèse A. Stukel

**Affiliations:** 1 Department of Medicine, University of British Columbia, British Columbia, 2775 Laurel Street, 10th Floor , Vancouver, V5Z 1M9 Canada; 2 ICES, 2075 Bayview Ave, Toronto, ON M4N 3M5 Canada; 3 Dalla Lana School of Public Health, University of Toronto, 155 College St, Toronto, ON M5T 3M7 Canada; 4 Institute of Health Policy, Management and Evaluation, University of Toronto, 155 College St, Toronto, ON M5T 3M7 Canada; 5 Department of Family and Community Medicine, University of Toronto, 500 University Ave, Toronto, ON M5G 1V7 Canada; 6 MAP Centre for Urban Health Solutions, St. Michael’s Hospital, 30 Bond Street, Toronto, ON M5B 1W8 Canada; 7 Dartmouth Institute for Health Policy & Clinical Practice, Geisel School of Medicine At Dartmouth, 74 College Street, Hanover, NH 03755 USA

**Keywords:** Dementia, Patient volume, Quality indicators, Primary health care

## Abstract

**Background:**

Some jurisdictions restrict primary care physicians’ daily patient volume to safeguard quality of care for complex patients. Our objective was to determine whether people with dementia receive lower-quality care if their primary care physician sees many patients daily.

**Methods:**

Population-based retrospective cohort study using health administrative data from 100,256 community-living adults with dementia aged 66 years or older, and the 8,368 primary care physicians who cared for them in Ontario, Canada. Multivariable Poisson GEE regression models tested whether physicians’ daily patient volume was associated with the adjusted likelihood of people with dementia receiving vaccinations, prescriptions for cholinesterase inhibitors, benzodiazepines, and antipsychotics from their primary care physician.

**Results:**

People with dementia whose primary care physicians saw ≥ 30 patients daily were 32% (95% CI: 23% to 41%, *p* < 0.0001) and 25% (95% CI: 17% to 33%, *p* < 0.0001) more likely to be prescribed benzodiazepines and antipsychotic medications, respectively, than patients of primary care physicians who saw < 20 patients daily. Patients were 3% (95% CI: 0.4% to 6%, *p* = 0.02) less likely to receive influenza vaccination and 8% (95% CI: 4% to 13%, *p* = 0.0001) more likely to be prescribed cholinesterase inhibitors if their primary care physician saw ≥ 30 versus < 20 patients daily.

**Conclusions:**

People with dementia were more likely to receive both potentially harmful and potentially beneficial medications, and slightly less likely to be vaccinated by high-volume primary care physicians.

**Supplementary Information:**

The online version contains supplementary material available at 10.1186/s12875-021-01398-9.

## Background

In 2015, 46 million people in the world had dementia; this number is expected to double every 20 years as the global population ages, reaching 131.5 million by 2050 [1]. Up to 70% of older adults with dementia live in private community dwellings [2] and the majority of their care is provided by primary care physicians (PCPs) [3, 4]. Caring for older adults with dementia is complex; most have numerous medical comorbidities and are more likely to experience adverse side effects to commonly prescribed medicines [5]. As the prevalence of dementia increases globally, PCPs and their governing bodies urgently need to understand how to structure primary care to provide older adults with dementia the care they require. Daily patient volume is a policy-sensitive aspect of primary care delivery [6]; some jurisdictions cap the number of patients PCPs may bill for seeing each day [7] and other regions are considering similar restrictions [8]. The effect of PCPs’ daily patient volume on quality of dementia care is unknown.

Quality primary care for people with dementia arises from judicious provision of helpful interventions and withholding or withdrawing potentially harmful ones. PCPs with high daily patient volumes are known to spend significantly less time (8.8 versus 12.5 min per visit) with each patient, [9] bill fewer service codes that require long visits, [10] and rely more heavily on non-physician staff to refill prescriptions [11] than low-volume PCPs. In keeping with Burgess’ cognitive burden framework, we hypothesized that these time pressures and organizational routines associated with increased daily patient volume would compromise PCP’s ability to partake in the “controlled processing” necessary to optimize care in complex patients with dementia [12]. This deleterious effect of high daily patient volume on quality of primary care has been demonstrated in the management of other conditions: high-volume PCPs inappropriately prescribe antibiotics to treat viral infections at a higher rate than their low-volume counterparts; [13, 14] they also provide lower-quality preventive care and medication management to people with asthma, angina, and diabetes than physicians who see fewer patients per day [6, 15, 16]. Even if high-volume PCPs were able to achieve controlled processing with reduced visit length, we posit that they would have less time to have the sometimes-difficult conversations around starting or stopping medications that target the neuropsychiatric symptoms of dementia [17]. 

Our main objective was to determine the association between PCP’s daily patient volume and provision of quality primary care to their patients with dementia. We examined annual vaccination against influenza and cholinesterase inhibitor prescriptions as indicators of good quality care, given their favourable risk–benefit ratio in most older adults with dementia [18, 19]. Benzodiazepine and antipsychotic prescriptions were selected as indicators of lower quality primary care because their adverse side-effect profile supports extremely stringent use in this population, [20] despite their indication in select patients with dementia [21]. Our secondary objective was to assess whether patients’ annual visit frequency, continuity of care, and long-term relationships with their PCP benefited patients of high-volume PCPs more than those of low-volume PCPs. Our hypothesis was that these features of the doctor-patient relationship might reduce cognitive burden in high-volume physicians and afford the controlled processing and communication time necessary to optimize complex patient care during brief office visits.

## Methods.

### Study Design and Setting

We conducted a population-based retrospective cohort study of 100,256 older adults with dementia and the 8,368 PCPs who cared for them. This study was set in Ontario, Canada, where medically necessary physician and hospital care is publicly insured for all residents, as are medications for adults 65 years of age and older. This manuscript is reported in accordance with the STROBE and RECORD statements for the reporting of observational studies conducted with health administrative data [22, 23].

### Data Sources

We created our cohort using datasets that were linked using unique encoded identifiers and analyzed at ICES. ICES is an independent, non-profit research institute whose legal status under Ontario’s health information privacy law allows it to collect and analyze health care and demographic data, without consent, for health system evaluation and improvement. The following Ontario health administrative databases were included: Registered Persons Database, Ontario Health Insurance Plan Database (OHIP), [24] Discharge Abstract Database, [25] Ontario Drug Benefit Database (ODB), [26] National Ambulatory Care Reporting System, [27] the rurality index of Ontario, [28] ICES Physician Database, [29] Client Agency Program Enrollment, and Ontario Marginalization Index, [30] and the Immigration, Refugees and Citizenship Canada Permanent Resident (IRCC-PR) database [31]. A description of these databases and their contents is available in Additional file 1.

### Study Population

**Fig. 1:**
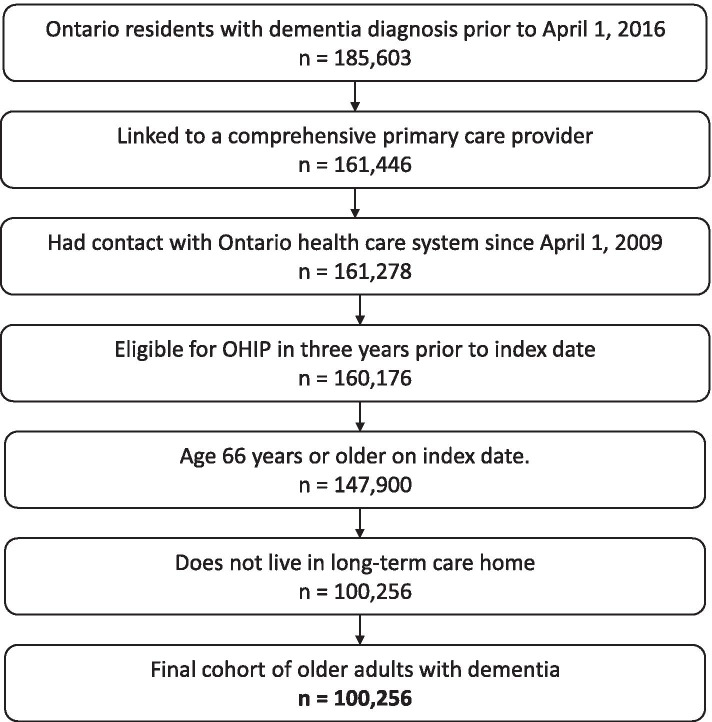
Study Flow Diagram

We enrolled people aged 66 years or older who had dementia as of April 1, 2016, the index study date, and received care from a comprehensive PCP [32]. Dementia diagnosis was determined using a validated algorithm with 79.3% sensitivity and 99.1% specificity [33]. Patients were excluded if they were not Ontario residents or did not have access to provincial health insurance in the previous three years, or were residents of long-term care homes (Fig. 1). Our observation period was one year, with a maximum follow-up date of March 31, 2017. Baseline characteristics and doctor-patient relationship variables were determined in the two years prior to index date, unless specified otherwise. PCPs were included if they were the designated PCP for at least one study participant [34] and met the criteria for a comprehensive PCP [32] in at least one year from April 1, 2013 to March 31, 2016.

### Measures

#### Outcomes.

We defined receipt of influenza vaccine using validated OHIP codes with 74.3% sensitivity and 73.8% specificity among people with dementia [35]. Filled prescriptions from patients’ PCPs for cholinesterase inhibitors, antipsychotic medications and benzodiazepines in the year following index date were identified from the ODB. See Additional file 2 for a complete list of diagnostic codes and drug names used to classify these study variables.

#### Exposure.

Patients were assigned to the PCP to whom they were rostered or virtually rostered [34]. The exposure of interest was the mean number of patients seen per day by the patient’s PCP (henceforth “daily patient volume”). Daily patient volume was calculated as the total number of unique office visits to a PCP (by all patients, not just those with dementia) between April 1, 2013 and March 31, 2016, divided by the total number of dates on which those visits took place [6]. We chose < 20, 20–24, 25–29 and ≥ 30 patients per day as a priori volume cut-offs, so that “high-volume” PCPs were those who saw 30 or more patients per day and “low-volume” PCPs saw fewer than 20 patients per day. We chose these cut-points to bracket the commonly quoted estimate that primary care physicians can reasonably care for about 25 patients per day [8, 36].

#### Doctor-Patient Relationship Variables

We also assessed whether the effects of doctor-patient relationship on outcomes was the same for patients of high- versus low-volume PCPs. These were: (a) high visit frequency, defined as seven or more visits per year to the PCP (the 75^th^ percentile in our sample) in the two years prior to the index date [37]; (b) high continuity of care, defined as patients seeing their PCP for 75% or more of all physician visits in the two years prior to index [38]; and (c) long doctor-patient relationship, defined as patients being attached to their PCP for five or more years [39]. Main effects of doctor-patient relationship variables were examined to facilitate interpretation of their interactions with daily patient volume.

#### Confounders

Patient characteristics adjusted for in multivariable models include age group-sex interactions, years since dementia diagnosis, CIHI Population Grouper comorbidity index [40] urban/rural location of residence, neighborhood deprivation quintile, and immigration to Canada in the past 10 years [31]. We also adjusted for outcome-specific variables such as past-year hospitalizations for chronic obstructive pulmonary disease (COPD) in influenza vaccine models. PCP variables adjusted for were age, sex, years since graduation, Canadian medical training, and urban/rural location of practice. Patient characteristics in the causal pathway (i.e., past-year medication and health care use, specialist visits) and PCP panel size and payment model were included in descriptive analyses but not regression models. Data on type of dementia (i.e., Alzheimer’s, vascular, etc.), were only available for persons who had been hospitalized in the years leading up to the index data, therefore these data were described but omitted from analyses.

### Statistical Analysis

The unit of analysis was the individual patient with dementia and the exposure was their PCP’s daily patient volume. All analyses were pre-specified based on our a priori hypotheses. We reported baseline characteristics according to daily patient volume groups. We estimated the confounder-adjusted relative rate of influenza vaccination, cholinesterase inhibitor prescription, benzodiazepine prescription and antipsychotic prescription separately using Poisson GEE models that accounted for clustering of patients within PCPs. Patients who died during follow-up were censored on the date of death. We also assessed main effects of doctor-patient relationship variables and their interactions with daily patient volume.

Secondary analyses examined the proportion of patients in each PCP volume group who changed physicians during the study period, as well as the effect of daily patient volume on medications prescribed by all physicians (not just PCPs). Multivariable analyses were completed using *proc gee* [41] in SAS version 9.04.

## Results

Our cohort consisted of 100,256 community-living older adults with dementia (Fig. 1), among whom 58,993 (59%) were female and 40,928 (41%) were 85 years or older. People were cared for by 8,368 comprehensive PCPs (45% female) who had a median panel size of 1,281 (IQR: 856–1,790) patients. The median daily patient volume was 23 (IQR: 18–31) patients per day (Table A, Additional file 3). Patients of high-volume PCPs (≥ 30 patients per day) were more likely to come from deprived neighborhoods and live in urban settings than patients whose PCPs saw fewer than 20 patients daily (Table 1). The number of unique medications taken at index date and past-year benzodiazepine and antipsychotic use were higher among patients of high-volume PCPs. There were no significant differences in proportion of people who had been treated by psychiatrists, geriatricians or neurologists in the year prior to index date, nor were there differences in past-year rates of hospitalizations or emergency department visits. People with dementia who had high-volume PCPs had an average of 13 visits per year to their PCP, as compared with eight visits/year among patients of low-volume PCPs. During the study year, 6,950 (6.9%) of people changed PCPs, with no differences in rate of switching across daily patient volume groups (Table B, Additional file 3).

**Table 1:** Baseline Characteristics of 100,256 Community-living Ontarians with Dementia, by PCP’s daily patient volume

	Average Number of Patients Seen Daily by Patient’s Primary Care Physician				
	< 20	20–24	25–29	≥ 30	Standardizessd Difference^a^ (< 20 vs ≥ 30)
Patient Characteristics	*N* = 36,150 (%)	*N* = 21,362 (%)	*N* = 15,224 (%)	*N* = 27,520 (%)	
Age group					
66–69	2,078 (5.7%)	1,160 (5.4%)	822 (5.4%)	1,659 (6.0%)	0.01
70–74	4,034 (11.2%)	2,270 (10.6%)	1,704 (11.2%)	3,310 (12.0%)	0.03
75–79	6,373 (17.6%)	3,800 (17.8%)	2,607 (17.1%)	4,960 (18.0%)	0.01
80–84	8,768 (24.3%)	5,310 (24.9%)	3,691 (24.2%)	6,782 (24.6%)	0.01
85 +	14,897 (41.2%)	8,822 (41.3%)	6,400 (42.0%)	10,809 (39.3%)	0.04
Sex					
Female	21,794 (60.3%)	12,598 (59.0%)	8,779 (57.7%)	15,822 (57.5%)	0.06
Male	14,356 (39.7%)	8,764 (41.0%)	6,445 (42.3%)	11,698 (42.5%)	0.06
Neighborhood Deprivation Index					
1—least deprived	6,422 (17.8%)	3,554 (16.6%)	2,480 (16.3%)	3,396 (12.3%)	0.15
2	7,104 (19.7%)	3,994 (18.7%)	2,822 (18.5%)	4,332 (15.7%)	0.10
3	7,065 (19.5%)	4,186 (19.6%)	2,868 (18.8%)	4,946 (18.0%)	0.04
4	7,296 (20.2%)	4,295 (20.1%)	3,154 (20.7%)	6,442 (23.4%)	0.08
5—most deprived	7,431 (20.6%)	4,886 (22.9%)	3,538 (23.2%)	7,618 (27.7%)	0.17
Missing data	832 (2.3%)	447 (2.1%)	362 (2.4%)	786 (2.9%)	0.03
Immigrated to Ontario in past 10 years	561 (1.6%)	346 (1.6%)	278 (1.8%)	753 (2.7%)	0.08
Location of dwelling					
Urban	25,141 (69.5%)	15,604 (73.0%)	11,533 (75.8%)	22,482 (81.7%)	0.29
Non-urban	7,328 (20.3%)	4,476 (21.0%)	3,034 (19.9%)	4,139 (15.0%)	0.14
Rural	3,478 (9.6%)	1,227 (5.7%)	615 (4.0%)	818 (3.0%)	0.28
Missing data	203 (0.6%)	55 (0.3%)	42 (0.3%)	81 (0.3%)	0.04
CIHI Comorbidity Group Index (2- year look-back)					
No health condition	328 (0.9%)	189 (0.9%)	142 (0.9%)	187 (0.7%)	0.03
Major and palliative	27,750 (76.8%)	16,028 (75.0%)	11,345 (74.5%)	19,888 (72.3%)	0.10
Moderate	5,193 (14.4%)	3,356 (15.7%)	2,387 (15.7%)	4,741 (17.2%)	0.08
Minor	2,570 (7.1%)	1,564 (7.3%)	1,130 (7.4%)	2,226 (8.1%)	0.04
Non-user	309 (0.9%)	225 (1.1%)	220 (1.4%)	478 (1.7%)	0.08
Type of Dementia					
Alzheimer’s	2,864 (7.9%)	1,665 (7.8%)	1,169 (7.7%)	2,100 (7.6%)	0.01
Vascular	786 (2.2%)	474 (2.2%)	309 (2.0%)	580 (2.1%)	0.00
Other	9,119 (25.2%)	5,484 (25.7%)	3,945 (25.9%)	6,896 (25.1%)	0.00
Missing data	23,381 (64.7%)	13,739 (64.3%)	9,801 (64.4%)	17,944 (65.2%)	0.01
Years since dementia diagnosis					
0–1	14,456 (40.0%)	8,190 (38.3%)	5,851 (38.4%)	10,158 (36.9%)	0.06
2–9	19,415 (53.7%)	11,744 (55.0%)	8,385 (55.1%)	15,316 (55.7%)	0.04
≥ 10	2,279 (6.3%)	1,428 (6.7%)	988 (6.5%)	2,046 (7.4%)	0.04
# unique medications in use on index date					
0–5	7,015 (19.4%)	4,011 (18.8%)	2,679 (17.6%)	4,534 (16.5%)	0.08
6–9	8,468 (23.4%)	4,851 (22.7%)	3,311 (21.7%)	5,538 (20.1%)	0.08
10–19	15,372 (42.5%)	9,254 (43.3%)	6,542 (43.0%)	12,128 (44.1%)	0.03
20 +	5,295 (14.6%)	3,246 (15.2%)	2,692 (17.7%)	5,320 (19.3%)	0.12
Medications taken in year prior to index date					
Cholinesterase inhibitor	14,942 (41.3%)	9,265 (43.4%)	6,729 (44.2%)	12,112 (44.0%)	0.05
Benzodiazepine	4,864 (13.5%)	3,234 (15.1%)	2,451 (16.1%)	4,989 (18.1%)	0.13
Antipsychotic	5,273 (14.6%)	3,319 (15.5%)	2,489 (16.3%)	5,114 (18.6%)	0.11
Patient Rostering to PCP					
Not rostered	1,728 (4.8%)	750 (3.5%)	759 (5.0%)	2,061 (7.5%)	0.11
Rostered	32,192 (89.1%)	19,052 (89.2%)	13,228 (86.9%)	22,998 (83.6%)	0.16
Virtually rostered	2,230 (6.2%)	1,560 (7.3%)	1,237 (8.1%)	2,461 (8.9%)	0.11
Physicians seen in year prior to index date					
PCP	31,059 (85.9%)	18,732 (87.7%)	13,053 (85.7%)	23,645 (85.9%)	0.00
Psychiatrist or geriatric psychiatrist	4,013 (11.1%)	2,055 (9.6%)	1,444 (9.5%)	2,617 (9.5%)	0.05
Geriatrician	7,833 (21.7%)	4,599 (21.5%)	3,393 (22.3%)	6,090 (22.1%)	0.01
Neurologist	5,205 (14.4%)	2,876 (13.5%)	2,122 (13.9%)	3,790 (13.8%)	0.02
Past-Year Hospitalizations or Emergency Department Visits	16,655 (46.1%)	9,567 (44.8%)	6,920 (45.5%)	12,377 (45.0%)	0.02
Doctor-Patient Relationship Variables^b^					
• Annual number of visits to PCP	8.24 ± 8.60	10.02 ± 9.96	10.84 ± 11.10	13.50 ± 15.03	0.43
• High annual PCP visit frequency^c^	6,181 (17.1%)	5,109 (23.9%)	4,183 (27.5%)	9,847 (35.8%)	0.21
• Continuity of care^d^	0.73 ± 0.33	0.78 ± 0.31	0.77 ± 0.32	0.76 ± 0.33	0.11
• High (≥ 0.75) continuity of care	22,755 (62.9%)	15,069 (70.5%)	10,570 (69.4%)	19,052 (69.2%)	0.13
• Relationship duration^e^	8.20 ± 7.15	9.86 ± 7.42	10.00 ± 7.61	9.31 ± 7.38	0.15
• Same PCP for ≥ 5 years	20,991 (58.1%)	14,128 (66.1%)	10,047 (66.0%)	17,603 (64.0%)	0.12

^a^ Standardised difference of means comparing people whose PCP sees < 20 patients daily to people whose PCP sees ≥ 30 patients daily

^b^ Measured in two years prior to index date, from April 1, 2014 to March 31, 2016

^c^ Patient made average of ≥ 7 visits/year between April 1, 2014 to March 31, 2016

^d^ Proportion of all physician visits from April 1, 2014 to March 31, 2016 that were to PCP

^¶^ Length of time in years patient cared for by current PCP

High-volume PCPs were significantly different from PCPs who saw fewer than 20 patients daily: their median panel size was more than twice as large (2,002 vs 977 patients) and they were more likely to be older, male, Canadian medical graduates, and practice in urban areas (Table 2). More than three quarters (75.3%) of high-volume PCPs were remunerated on a primarily fee-for-service basis, compared to only 25.6% of low-volume PCPs.

**Table 2:** Characteristics of Primary Care Physicians (PCPs) Caring for Older Ontarians with Dementia

	Average Number of Patients Seen Daily by Primary Care Physicians				Standardized Difference (< 20 vs ≥ 30)
	< 20	20 -24	25–29	≥ 30	
Physician Characteristics	*N* = 3,884 (%)	*N* = 1,662 (%)	*N* = 1,059 (%)	*N* = 1,763 (%)	
Panel Size					
Mean ± SD	988 ± 491	1,425 ± 562	1,559 ± 646	2,035 ± 986	1.34
Median (IQR)	977 (688–1,299)	1,452 (1,094–1,805)	1,598 (1,118–2,018)	2,002 (1,385–2,597)	
Physician age					
Mean ± SD	49.9 ± 12.9	53.2 ± 11.9	54.0 ± 12.0	54.3 ± 11.0	0.36
Median (IQR)	50 (39–60)	54 (45–62)	55 (45–64)	54 (46–62)	
Physician sex					
Female	2,259 (58.2%)	691 (41.6%)	355 (33.5%)	469 (26.6%)	0.67
Male	1,619 (41.7%)	967 (58.2%)	702 (66.3%)	1,291 (73.2%)	0.67
Missing data	6 (0.2%)	< = 5 (0.2%)	< = 5 (0.2%)	< = 5 (0.2%)	N/A^a^
Years since medical school graduation					
≤ 15	1,358 (35.0%)	353 (21.2%)	208 (19.6%)	250 (14.2%)	0.50
16–25	854 (22.0%)	387 (23.3%)	243 (22.9%)	494 (28.0%)	0.14
26–35	966 (24.9%)	511 (30.7%)	281 (26.5%)	531 (30.1%)	0.12
≥ 36	699 (18.0%)	407 (24.5%)	325 (30.7%)	485 (27.5%)	0.23
Missing data	7 (0.2%)	< = 5 (0.2%)	< = 5 (0.2%)	< = 5 (0.2%)	N/A^a^
Canadian medical graduate					
Yes	2,913 (75.0%)	1,103 (66.4%)	619 (58.5%)	842 (47.8%)	0.58
No	693 (17.8%)	465 (28.0%)	394 (37.2%)	864 (49.0%)	0.70
Missing data	278 (7.2%)	94 (5.7%)	46 (4.3%)	57 (3.2%)	0.18
Practice location					
Urban	2,662 (68.5%)	1,203 (72.4%)	815 (77.0%)	1,463 (83.0%)	0.34
Non-urban	600 (15.4%)	291 (17.5%)	168 (15.9%)	204 (11.6%)	0.11
Rural	295 (7.6%)	72 (4.3%)	24 (2.3%)	35 (2.0%)	0.26
Missing data	327 (8.4%)	96 (5.8%)	52 (4.9%)	61 (3.5%)	0.21
Payment model					
Fee-for-service (FFS)^b^	403 (10.4%)	132 (7.9%)	97 (9.2%)	255 (14.5%)	0.12
Enhanced FFS^c^					
CCM	87 (2.2%)	55 (3.3%)	57 (5.4%)	127 (7.2%)	0.24
FHG	504 (13.0%)	447 (26.9%)	391 (36.9%)	945 (53.6%)	0.96
Capitation^d^					
FHT	1,589 (40.9%)	412 (24.8%)	164 (15.5%)	109 (6.2%)	0.90
FHN or FHO	1,221 (31.4%)	609 (36.6%)	349 (33.0%)	327 (18.5%)	0.30
Other	80 (2.1%)	7 (0.4%)	< = 5 (0.1%)	0 (0.0%)	0.21
Works Fulltime (FTE ≥ 1.00)	1,665 (42.9%)	1,434 (86.3%)	984 (92.9%)	1,692 (96.0%)	1.41

^a^ N/A: Could not report SDs comparing cell sizes that are too small to report while maintaining confidentiality

^b^ Fee-for-service: Income through fee for service billings. How PCPs not in payment enrollment or salary model

^c^ Enhanced fee-for-service: Majority of income through fee for service billings with incentives for chronic disease management and preventive care. Includes: CCM: Comprehensive Care Mode, FHG: Family Health Group

^d^ Capitation and blended capitation models: Majority of income through capitation fees. Capitation based on a defined basket of primary care services provided to enrolled patients based on age/sex of each patient. Fee-for-service paid for other services. Additionally, physicians receive monthly comprehensive care capitation payments for all enrolled patients. Includes: FHT: Family Health Team, FHN: Family Health Network, FHO: Family Health Organization

In our primary analysis (Table 3), older adults with dementia were 1.32 (95% CI: 1.23 to 1.41) and 1.25 (95% CI: 1.17 to 1.33) times more likely to receive benzodiazepine and antipsychotic prescriptions from high-volume PCPs than low-volume PCPs (Table 3; full model in Table D, Additional file 3). They were also more likely (aRR 1.08, 95% CI:1.04 to 1.13) to receive cholinesterase inhibitor prescriptions and slightly less likely (aRR 0.97, 95% CI: 0.94 to 0.996) to be vaccinated against influenza than people with dementia cared for by low-volume PCPs. Patients received most prescriptions for cholinesterase inhibitors, benzodiazepines and antipsychotics from their PCP (Table C, Additional file 3) and the magnitude of the volume-outcome relationships was diminished significantly when we included prescriptions from all physicians (Table E, Additional file 3).

**Table 3:** Adjusted Relative Rates of Vaccination and Prescriptions for People with Dementia, by PCP’s daily patient volume

Outcome	Influenza Vaccination		Cholinesterase Inhibitor Prescription		Benzodiazepine Prescription		Antipsychotic Prescription	
	Adjusted^a^ RR (95% CI)	*p*-value	Adjusted^a^ RR (95% CI)	*p*-value	Adjusted^a^ RR (95% CI)	*p*-value	Adjusted^a^ RR (95% CI)	*p*-value
Physician’s Daily Patient Volume								
< 20 patients/day	1.0 (reference)	-	1.0 (reference)	-	1.0 (reference)	-	1.0 (reference)	-
20 -24 patients/day	1.00 (0.98 to 1.03)	0.81	1.10 (1.05 to 1.14)	< .0001	1.17 (1.10 to 1.24)	< .0001	1.12 (1.05 to 1.19)	0.001
25–29 patients/day	0.98 (0.95 to 1.02)	0.32	1.09 (1.04 to 1.14)	0.0002	1.17 (1.09 to 1.26)	< .0001	1.15 (1.06 to 1.23)	0.0003
≥ 30 patients/day	0.97 (0.94 to 0.996)	0.02	1.08 (1.04 to 1.13)	0.0001	1.32 (1.23 to 1.41)	< .0001	1.25 (1.17 to 1.33)	< .0001

^a^All models were adjusted for the following confounders: patient age, sex, years since dementia diagnosis, CIHI Population Grouper category, urban/rural location of residence, neighborhood deprivation quintile, past 10-year immigration, and PCP age, sex, years since graduation, Canadian medical graduate, urban/rural location of practice. Models of influenza vaccination were also adjusted for past-year hospitalization for COPD

High visit frequency and high continuity of care were associated with an increased likelihood of influenza vaccination and higher levels of prescribing of all medications, adjusting for physician volume (Table 4). Patients who had known their doctor for at least five years were more likely to receive influenza vaccination and cholinesterase inhibitor prescriptions, but less likely (aRR 0.89, 95%CI: 0.85 to 0.94) to be prescribed antipsychotic medications, adjusting for daily patient volume and confounders. There were no significant interactions between the daily patient volume and the doctor-patient relationship variables with respect to prescribing of benzodiazepines or antipsychotics. There was a significant interaction between physician volume and both patient visit frequency and continuity of care, serving to increase the relative provision of cholinesterase inhibitors and influenza vaccinations by high compared to low volume physicians by about 8–15% (Table F, Additional file 3).

**Table 4:** Adjusted Main Effects of Doctor-Patient Relationship variables on Vaccination and Medication Receipt in Older Ontarians with Dementia

Effects	Influenza Vaccination		Cholinesterase Inhibitor Prescription		Benzodiazepine Prescription		Antipsychotic Prescription	
	Adjusted RR^a^ (95% CI)	*p*-value	Adjusted RR (95% CI)	*p*-value	Adjusted RR (95% CI)	*p*-value	Adjusted RR (95% CI)	*p*-value
High visit frequency (> 7 visits/year)	1.32 (1.30 to 1.34)	< .0001	1.09 (1.07 to 1.12)	< .0001	1.74 (1.67 to 1.82)	< .0001	1.25 (1.19 to 1.30)	< .0001
High continuity of care	1.19 (1.16 to 1.21)	< .0001	1.47 (1.43 to 1.52)	< .0001	1.42 (1.35 to 1.49)	< .0001	1.31 (1.24 to 1.37)	< .0001
Long (≥ 5 years) doctor-patient relationship	1.14 (1.11 to 1.17)	< .0001	1.13 (1.10 to 1.16)	< .0001	1.04 (0.996 to 1.10)	0.08	0.89 (0.85 to 0.94)	< .0001

Notes: ^a^All models were adjusted for the following confounders: patient age, sex, years since dementia diagnosis, CIHI Population Grouper category, urban/rural location of residence, neighborhood deprivation quintile, past 10-year immigration, and PCP daily patient volume, age, sex, years since graduation, Canadian medical graduate, urban/rural location of practice. Models of influenza vaccination were also adjusted for past-year hospitalization for COPD

## Discussion

In keeping with our cognitive burden hypothesis, we found that – controlling for morbidity and time since dementia diagnosis – community-dwelling people with dementia were more likely to receive both potentially harmful (benzodiazepine and antipsychotic) and potentially beneficial (cholinesterase inhibitor) prescriptions from their PCP if that physician saw ≥ 30 versus < 20 patients per day. Factors that increased face-time between doctors and their patients with dementia (i.e., more frequent visits, high continuity of care) were also associated with significantly increased likelihood of both desirable and undesirable prescribing, independent of daily patient volume. In contrast, patients of high-volume physicians were less likely to receive preventive care in the form of influenza vaccinations. Patients of high-volume physicians benefited more significantly than those of low-volume physicians from frequent office visits and high continuity of care when it came to cholinesterase inhibitor prescribing and vaccinations, but not benzodiazepine and antipsychotic prescribing.

Our “high volume” cut-off of 30 is lower than “high volume” definitions used in other studies (> 34 to ≥ 75 patients per day [6, 13, 14], as well as the daily patient caps (up to 65 patients per day) [7] that have been implemented in other jurisdictions. Even so, our findings are aligned with extant evidence that shows reduced preventive care in persons with diabetes by high-volume primary care providers, [6, 15, 16] as well as a correlation between visit frequency, continuity of care and likelihood of receiving preventive testing and medicines in persons with diabetes [37, 42] and heart disease. [43, 44] Similar to our findings, Cadieux et al. showed that high-volume PCPs were more likely than low-volume PCPs to inappropriately prescribe non-indicated antibiotics, [13] and Kroll et al. showed that frequent visitors in primary care were more likely to receive benzodiazepine prescriptions [45].

Although some high-volume PCPs work longer hours to see more patients per day, at least some of their high volume comes at the cost of visit-time per patient [10]. Benzodiazepines and antipsychotics are often prescribed to treat symptoms that are highly disruptive to people with dementia and their caregivers: sleep disturbance and other neuropsychiatric symptoms of dementia. In the face of such symptoms, the decision to withhold or discontinue treatment may require extensive discussion with patients and caregivers that high-volume physicians do not have time for [46]. Increased visit frequency and continuity of care may allow increased opportunity for primary care physicians to provide preventive care (i.e., cholinesterase inhibitors and vaccinations), but may also increase the number of times physicians are faced with caregivers and patients requesting medications to cope with distressing symptoms [47].

Our study has some limitations. We did not have data on number of hours worked by physicians; therefore, we are unable to directly assess whether shorter visit length was driving differences in outcomes between high- versus low-volume PCPs. There is, however, significant existing evidence that high-volume PCPs achieve their high volume through briefer appointments [9–11]. Our use of health administrative data limited the scope of primary care quality indicators for people with dementia we could assess; the relation of high-volume care and quality indicators such as patient or caregiver satisfaction, frequent reassessment of cognitive status and provision of caregiver support [20, 48, 49] should be studied in subsequent work. Also, our measure of influenza vaccination has only moderate sensitivity and specificity. [35] We were only able to describe dementia type among 35% of our sample and have no data on specific neuropsychiatric symptoms and their severity for each patient. The balance of risks and benefits associated with prescription of medications to patients with dementia is determined in part by these clinical features; [50] therefore, there is some inherent misclassification of these processes when identifying them as either desirable or undesirable at a population level. Finally, patients of high-volume physicians in our study had higher rates of benzodiazepine and antipsychotic prescribing in the year prior to the study start. Although differences in baseline prescribing could explain ongoing differences in prescribing, we argue that PCPs are responsible for both initiating beneficial medications and deprescribing inappropriate medications in their patients. Given the absence of differences in all available measures of morbidity across daily patient volume groups (type/duration of dementia diagnosis, past-year hospitalizations and emergency department visits, CIHI Population Grouper comorbidity index), persistence of these prescribing differences may reflect ineffective deprescribing of potentially inappropriate medications among high-volume physicians.

A strength of our study is its inclusion of all older adults with dementia and the PCPs who care for them in Ontario’s single-payer health care system. We also defined daily patient volume a priori, based on cut-points that have been proposed as thresholds of safe care.[8] Our examination of four process outcomes provides a balanced perspective on how high daily patient volume affects quality of care and our sensitivity analyses illustrate that our results are robust.

We found that high-volume primary care physicians take care of much larger panels of more deprived patients in urban settings than low-volume physicians. Although daily patient volume caps may improve some aspects of care for persons with dementia, they may not be feasible in rural districts where high volume is necessary to afford timely access to care. Development and evaluation of tools and practice models that facilitate optimal care for complex patients during rushed office visits will therefore be critical in the coming years. Similarly, models of care that reduce the significant burden of non-clinical work on physicians’ daily schedules should also be investigated and supported so that family doctors may spend a larger fraction of their time caring for complex patients with dementia and other chronic diseases. The association of daily patient volume with other outcomes that can be assessed in administrative data – such as pneumococcus vaccination, fall-related injuries, long-term care home admission and death – should also be examined in subsequent studies to further interrogate the volume-quality relationship identified here.

## Conclusion

As the global population of people with dementia grows, evidence is needed to support optimization of primary care for this complex patient population. This study shows that people with dementia are more likely to receive both potentially harmful and potentially beneficial prescriptions, and slightly less likely to receive beneficial vaccinations if their primary care physician sees many patients per day. Primary care physicians’ daily patient volume should be considered by practitioners and policymakers seeking to affect change in quality of dementia care. Impact of daily patient volume restrictions on other aspects of care – such as access – requires ongoing study.

## Authors’ information

NEL is a resident physician in Internal Medicine at the University of British Columbia, and a post-doctoral fellow in Primary Care and Health Systems at ICES Toronto. VL is an analyst at ICES Toronto. RHG is Scientific Director of the Institute of Health Services and Policy Research at the Canadian Institutes of Health Research (CIHR) and a Senior Core Scientist at ICES Toronto. He is also a staff family physician at St. Michael’s Hospital in Toronto and a Scientist in its Centre for Urban Health Solutions, a Professor at the University of Toronto in the Department of Family and Community Medicine, Faculty of Medicine, and Dalla Lana School of Public Health. TAS is a Canadian statistician who works as a Senior Core Scientist at ICES Toronto, as a professor in the Institute of Health Policy, Management and Evaluation of the University of Toronto, and as an adjunct professor of epidemiology and of The Dartmouth Institute for Health Policy and Clinical Practice at Dartmouth College.

## Supplementary Information

**Additional file 1.** Data sources.

**Additional file 2.** Variable Coding.

**Additional file 3.** Additional Data & analyses.

## Data Availability

The individual-level data underlying this study are based on records generated from the administration of Ontario’s publicly funded health system. ICES has a special designation under Ontario’s Personal Health Information Protection Act to use these data in studies that evaluate health care delivery and outcomes. This designation is granted by the Information and Privacy Commissioner of Ontario, and is contingent on a triennial review and ongoing oversight of the privacy practices at ICES. A variety of measures are deployed to protect the personal health information entrusted to ICES and, under the Personal Health Information Protection Act (Ontario Regulation 329/04), the underlying data are legally not allowed for public repository. The data that support the findings of this study are available from ICES but restrictions apply to the availability of these data, which were used under license for the current study, and so are not publicly available. Data are however available from the authors upon reasonable request and with permission of ICES.

## Abbreviations

aRR: Adjusted relative risk
COPD: Chronic obstructive pulmonary disease
OHIP: Ontario Health Insurance Plan Database
ODB: Ontario Drug Benefit Database
PCP: Primary care physician
RECORD: Reporting of Studies Conducted Using Observational Routinely-collected Data
STROBE: Strengthening the Reporting of Observational Studies in Epidemiology
