# Supplementary material for: Primary care physician volume and quality of care for older adults with dementia: a retrospective cohort study
Source: BMC Fam Pract. 2021 Mar 9;22:51. doi: 10.1186/s12875-021-01398-9 (PMC7945328; doi:10.1186/s12875-021-01398-9)
Supplement: Supplementary file 2 — Additional file 2. Variable Coding. [file 12875_2021_1398_MOESM2_ESM.docx]

**Primary care physician volume and quality of care for older adults with dementia: a retrospective cohort study**

**Authors**:

Natasha E. Lane*^a,b^ MD, PhD natasha.lane@alumni.ubc.ca

Vicki Ling^b^ MSc vicki.ling@ices.on.ca

Richard H. Glazier^b,c,d,e,f^ MD, MPH rick.glazier@ices.on.ca

Thérèse A. Stukel^b,d,g^, PhD therese.stukel@ices.on.ca

# Additional File 2: Variable Coding

| **Variable** | **Code** |
| --- | --- |
| Daily patient volume | From OHIP. For each eligible PCP physician, calculate the total number of unique office visits (by all patients, not just patients in disease cohort) between April 1 2013 and March 31 2016. (Location in (‘O’, ‘L’, ‘H’, ‘P’). Divide by the total number of dates on which those visits took place. Exclude: any days with 4 or fewer visits because not typical ambulatory practice days. Result is mean number of patients seen per day worked. |
| Influenza Vaccination | From OHIP. One of following coded in 1-year post-index date. OHIP ICD-9-CA Service Code: G590 or G591 or Q130. ODB DIN: 2362384, 2420783, 2269562, 2426544, 2420686, 2420643, 2428881, 2432730 |
| Cholinesterase Inhibitor or memantine prescription | From ODB, linked with PCP. Prescriptions filled from PCP for any of the following medications in year following index date:  Donepezil HCL, Galantamine, Rivastigmine, Rivastigmine tartate Memantine HCL |
| Benzodiazepine prescription | From ODB, linked with PCP. Prescriptions filled from PCP for any of the following medications in year following index date:  Alprazolam, Bromazepam, Chlordiazepoxide, Chlordiazepoxide HCL, Chlordiazepoxide HCL & Clidinium HCL, Clobazam, Clonazepam, Clorazepate dipotassium, Diazepam, Diazepam & Methycellulose, Estazolam, Flurazepam, Flurazepam HCL, Ketazolam, Lorazepam, Midazolam, Midazolam HCL, Nitrazepam, Oxazepam, Temazepam, Triazolam |
| Antipsychotic prescription | From ODB, linked with PCP. Prescriptions filled from PCP for any of the following medications in year following index date:  Aripiprazole, Asenapine, Chlormezanone, Chlormezanone HCL, Chlorprothixene, Clozapine, Flupentixol, Flupentixol decanoate, Fluphenazine, Fluphenazine decanoate, Fluphenazine HCL, Fluspiriline, haloperidol, Haloperidol decanoate, Haloperidol decanoate, Loxapine, Loxapine HCL, Loxapine succinate, Mesoridazine besylate, Methotrimeprazine, Methotrimeprazine HCL, Methotrimeprazine maleate, Olanzapine, Paliperidone, Paliperidone palmitate, Periciazine, Perphenazine, Pumozide, Pipotiazine palmitate, Prochlorperazine, Prochlorperazine mesylate, Quetiapine fumarate, Risperidone, Risperidone tartrate, Thiopropazate HCL, Thiopropazate HCL, Thiopropazate mesylate, Thioridazine HCL, Thiothixene, Trifluoperazine HCL, Ziprasidone HCL, Zuclopenthixol acetate, Zuclopenthixol decanoate, Zuclopenthixol HCL. |

Note: Contact [Natasha.Lane@alumni.ubc.ca](mailto:Natasha.Lane@alumni.ubc.ca) for specific DINs associated with each drug listed above, as well as any related code used in this study.
