# Supplementary material for: Primary care physician volume and quality of care for older adults with dementia: a retrospective cohort study
Source: BMC Fam Pract. 2021 Mar 9;22:51. doi: 10.1186/s12875-021-01398-9 (PMC7945328; doi:10.1186/s12875-021-01398-9)
Supplement: Supplementary file 3 — Additional file 3. Additional Data & analyses. [file 12875_2021_1398_MOESM3_ESM.docx]

**Primary care physician volume and quality of care for older adults with dementia: a retrospective cohort study**

**Authors**:

Natasha E. Lane*^a,b^ MD, PhD natasha.lane@alumni.ubc.ca

Vicki Ling^b^ MSc vicki.ling@ices.on.ca

Richard H. Glazier^b,c,d,e,f^ MD, MPH rick.glazier@ices.on.ca

Thérèse A. Stukel^b,d,g^, PhD therese.stukel@ices.on.ca

# Additional File 3: Additional Data & Analyses

## Table A: Quantiles of Daily Patient Volume Among 8,368 Primary Care Physicians (PCPs) taking care of 100,256 Community-Living People with Dementia

| **Quantile** | **Daily Patient Volume** |
| --- | --- |
| **100% (Max)** | 112 |
| **99%** | 68 |
| **95%** | 50 |
| **90%** | 41 |
| **75%** | 31 |
| **50% (Median)** | 23 |
| **25%** | 18 |
| **10%** | 14 |
| **5%** | 12 |
| **1%** | 10 |
| **0% (Min)** | 5 |

## Table B: Proportion of 100,256 Community-living Ontarians with Dementia who stayed with versus changed Primary Care Physicians (PCPs) between April 1, 2016 and March 31, 2017.

|  | **Average Number of Patients Seen Daily by Patient’s Primary Care Physician** | | | |  |  |
| --- | --- | --- | --- | --- | --- | --- |
|  | **<20** | **20 -24** | **25-29** | **≥30** | **Standardized Difference***  **(<20 vs ≥30)** | |
|  | N=36,150  (%) | N=21,362  (%) | N=15,224  (%) | N=27,520  (%) |  | |
| - Stayed with same PCP | 31,438 (87.0%) | 18,943 (88.7%) | 13,418 (88.1%) | 24,418 (88.7%) | 0.05 | |
| - Switched to another PCP | 2,857 (7.9%) | 1,394 (6.5%) | 1,035 (6.8%) | 1,664 (6.0%) | 0.07 | |
| - Missing data | 1,855 (5.1%) | 1,025 (4.8%) | 771 (5.1%) | 1,438 (5.2%) | 0.004 | |

## Table C: Influenza Vaccination and Type of Physician Prescribing Cholinesterase Inhibitors, Benzodiazepines and Antipsychotic Medications to Older Ontarians with Dementia from April 1, 2016 to March 31, 2017, by daily patient volume

|  | **Average Number of Patients Seen Daily by Primary Care Physicians** | | | |
| --- | --- | --- | --- | --- |
|  | **<20** | **20 -24** | **25-29** | **≥30** |
| **Cholinesterase inhibitors** |  |  |  |  |
| - Prescriptions from any physician | 14,853 (41.1%) | 9,159 (42.9%) | 6,596 (43.3%) | 11,672 (42.4%) |
| - Had ≥1 prescription by own PCP | 10,164 (28.1%) | 6,645 (31.1%) | 4,633 (30.4%) | 8,129 (29.5%) |
| - Had ≥1 prescription by GP/FP in PCP’s practice group | 10,600 (29.3%) | 6,810 (31.9%) | 4,743 (31.2%) | 8,311 (30.2%) |
| - Had ≥ 1 by other GP/FP, none from own PCP’s practice group | 1,753 (4.8%) | 953 (4.5%) | 773 (5.1%) | 1,428 (5.2%) |
| - No prescriptions from GP/FP, had ≥ 1 from a specialist | 2,106 (5.8%) | 1,235 (5.8%) | 980 (6.4%) | 1,752 (6.4%) |
| - All prescriptions' prescriber info missing | 394 (1.1%) | 161 (0.8%) | 100 (0.7%) | 181 (0.7%) |
| **Benzodiazepines** |  |  |  |  |
| - Prescriptions from any physician | 5,010 (13.9%) | 3,303 (15.5%) | 2,439 (16.0%) | 4,857 (17.6%) |
| - Had ≥1 prescription by own PCP | 3,116 (8.6%) | 2,229 (10.4%) | 1,591 (10.5%) | 3,316 (12.0%) |
| - Had ≥1 prescription by GP/FP in PCP’s practice group | 3,307 (9.1%) | 2,337 (10.9%) | 1,654 (10.9%) | 3,408 (12.4%) |
| - Had ≥ 1 by other GP/FP, none from own PCP’s practice group | 1,154 (3.2%) | 671 (3.1%) | 557 (3.7%) | 1,041 (3.8%) |
| - No prescriptions from GP/FP, had ≥ 1 from a specialist | 489 (1.4%) | 273 (1.3%) | 214 (1.4%) | 388 (1.4%) |
| - All prescriptions' prescriber info missing | 60 (0.2%) | 22 (0.1%) | 14 (0.1%) | 20 (0.1%) |
| **Antipsychotics** |  |  |  |  |
| - Prescriptions from any physician | 7,210 (19.9%) | 4,415 (20.7%) | 3,330 (21.9%) | 6,318 (23.0%) |
| - Had ≥1 prescription by own PCP | 3,847 (10.6%) | 2,569 (12.0%) | 1,855 (12.2%) | 3,592 (13.1%) |
| - Had ≥1 prescription by GP/FP in PCP’s practice group | 4,130 (11.4%) | 2,703 (12.7%) | 1,956 (12.8%) | 3,740 (13.6%) |
| - Had ≥ 1 by other GP/FP, none from own PCP’s practice group | 1,935 (5.4%) | 1,070 (5.0%) | 843 (5.5%) | 1,544 (5.6%) |
| - No prescriptions from GP/FP, had ≥ 1 from a specialist | 966 (2.7%) | 564 (2.6%) | 472 (3.1%) | 919 (3.3%) |
| - All prescriptions' prescriber info missing | 179 (0.5%) | 78 (0.4%) | 59 (0.4%) | 115 (0.4%) |
| **Influenza Vaccination** | 18,024 (49.9%) | 10,588 (49.6%) | 7,308 (48.0%) | 12,845 (46.7%) |

Notes: GP/FP = General Practitioner or Family Physician

## Table D: Complete Results of Multivariable Poisson GEE to Assess Effect of Primary Care Providers’ Daily Patient Volume on Vaccinations and Medications and Given to Older Ontarians with Dementia (n = 100, 256)

| **Model** | **Influenza Vaccination** | | **Cholinesterase Inhibitor Prescription** | | **Benzodiazepine Prescription** | | **Antipsychotic Prescription** | |
| --- | --- | --- | --- | --- | --- | --- | --- | --- |
|  | **Adjusted RR**  **(95% CI)** | ***p*-value** | **Adjusted RR**  **(95% CI)** | ***p*-value** | **Adjusted RR**  **(95% CI)** | ***p*-value** | **Adjusted RR**  **(95% CI)** | ***p*-value** |
| **Physician’s Daily Patient Volume** |  |  |  |  |  |  |  |  |
| <20 patients/day | Reference | **-** | Reference | **-** | Reference | **-** | Reference | **-** |
| 20 -24 patients/day | 1.00  (0.98 to 1.03) | 0.81 | 1.10  (1.05 to 1.14) | <.0001 | 1.17  (1.10 to 1.24) | <.0001 | 1.12  (1.05 to 1.19) | 0.001 |
| 25-29 patients/day | 0.98  (0.95 to 1.02) | 0.32 | 1.09  (1.04 to 1.14) | 0.0002 | 1.17  (1.09 to 1.26) | <.0001 | 1.15  (1.06 to 1.23) | 0.0003 |
| ≥30 patients/day | 0.97  (0.94 to 1.00) | 0.02 | 1.08  (1.04 to 1.13) | 0.0001 | 1.32  (1.23 to 1.41) | <.0001 | 1.25  (1.17 to 1.33) | <.0001 |
| **Patient Characteristics** |  |  |  |  |  |  |  |  |
| **Age group** |  |  |  |  |  |  |  |  |
| Females 66-69 | Reference | **-** | Reference | **-** | Reference | **-** | Reference | **-** |
| Females 70-74 | 1.01  (0.97 to 1.06) | 0.53 | 1.24  (1.14 to 1.35) | <.0001 | 0.86  (0.77 to 0.95) | 0.005 | 1.01  (0.90 to 1.14) | 0.86 |
| Females 75-79 | 1.02  (0.98 to 1.06) | 0.33 | 1.50  (1.39 to 1.62) | <.0001 | 0.83  (0.75 to 0.92) | 0.004 | 0.98  (0.88 to 1.10) | 0.76 |
| Females 80-84 | 1.00  (0.96 to 1.04) | 0.89 | 1.58  (1.46 to 1.70) | <.0001 | 0.77  (0.70 to 0.85) | <.0001 | 0.98  (0.88 to 1.09) | 0.74 |
| Females 85+ | 0.89  (0.86 to 0.93) | <.0001 | 1.39  (1.29 to 1.50) | <.0001 | 0.71  (0.65 to 0.79) | <.0001 | 1.10  (0.99 to 1.22) | 0.08 |
| Males 66-69 | 1.00  (0.95 to 1.05) | 0.88 | 0.79  (0.71 to 0.88) | <.0001 | 0.79  (0.70 to 0.90) | 0.001 | 0.88  (0.76 to 1.02) | 0.08 |
| Males 70-74 | 1.06  (1.01 to 1.11) | 0.01 | 1.13  (1.04 to 1.23) | 0.004 | 0.71  (0.63 to 0.79) | <.0001 | 0.95  (0.84 to 1.07) | 0.41 |
| Males 75-79 | 1.10  (1.06 to 1.15) | <.0001 | 1.42  (1.31 to 1.53) | <.0001 | 0.57  (0.51 to 0.64) | <.0001 | 0.88  (0.78 to 0.99) | 0.03 |
| Males 80-84 | 1.09  (1.05 to 1.14) | <.0001 | 1.52  (1.41 to 1.63) | <.0001 | 0.57  (0.51 to 0.63) | <.0001 | 0.92  (0.82 to 1.03) | 0.15 |
| Males 85+ | 0.96  (0.92 to 1.00) | 0.05 | 1.44  (1.33 to 1.55) | <.0001 | 0.53  (0.47 to 0.58) | <.0001 | 0.91  (0.82 to 1.02) | 0.11 |
| **Neighborhood Deprivation Index** |  |  |  |  |  |  |  |  |
| 1 - least deprived | Reference | - | Reference | - | Reference | - | Reference |  |
| 2 | 0.94  (0.92 to 0.97) | <.0001 | 0.97  (0.94 to 1.00) | 0.07 | 0.99  (0.93 to 1.06) | 0.82 | 1.07  (1.00 to 1.14) | 0.05 |
| 3 | 0.94  (0.92 to 0.96) | <.0001 | 0.95  (0.92 to 0.98) | 0.003 | 1.01  (0.94 to 1.07) | 0.86 | 1.03  (0.97 to 1.11) | 0.35 |
| 4 | 0.94  (0.91 to 0.96) | <.0001 | 0.96  (0.92 to 0.99) | 0.01 | 1.08  (1.01 to 1.16) | 0.02 | 1.07  (1.00 to 1.14) | 0.05 |
| 5 - most deprived | 0.91  (0.89 to 0.93) | <.0001 | 0.89  (0.86 to 0.92) | <.0001 | 1.14  (1.07 to 1.21) | <.0001 | 1.06  (1.00 to 1.14) | 0.06 |
| Missing data | 0.71  (0.66 to 0.75) | <.0001 | 0.86  (0.80 to 0.93) | <.0001 | 0.85  (0.73 to 0.99) | 0.03 | 0.97  (0.85 to 1.10) | 0.63 |
| **Immigrated to Ontario in past 10 years** |  |  |  |  |  |  |  |  |
| No | Reference | **-** | Reference | **-** | Reference | **-** | Reference | **-** |
| Yes | 0.82  (0.78 to 0.87) | <.0001 | 0.93  (0.86 to 1.01) | 0.08 | 0.82  (0.71 to 0.96) | 0.01 | 0.92  (0.80 to 1.06) | 0.24 |
| **Location of dwelling** |  |  |  |  |  |  |  |  |
| urban | Reference | **-** | Reference | - | Reference | - | Reference | - |
| non-urban | 1.0  (0.97 to 1.03) | 0.88 | 1.11  (1.07 to 1.16) | <.0001 | 1.05  (0.98 to 1.14) | 0.18 | 1.11  (1.02 to 1.20) | 0.02 |
| rural | 0.98  (0.93 to 1.02) | 0.32 | 1.17  (1.10 to 1.25) | <.0001 | 1.02  (0.91 to 1.16) | 0.69 | 1.07  (0.95 to 1.20) | 0.29 |
| Missing data | 0.80  (0.70 to 0.92) | 0.001 | 1.04  (0.89 to 1.22) | 0.60 | 0.91  (0.65 to 1.29) | 0.61 | 0.80  (0.57 to 1.13) | 0.20 |
| **CIHI Comorbidity Group Index (2- year look-back)** |  |  |  |  |  |  |  |  |
| no health condition | Reference | **-** | Reference | - | Reference | - | Reference | - |
| major and palliative | 1.59  (1.43 to 1.77) | <.0001 | 1.04  (0.94 to 1.15) | 0.44 | 1.82  (1.39 to 2.38) | <.0001 | 1.53  (1.24 to 1.88) | <.0001 |
| moderate | 1.81  (1.62 to 2.02) | <.0001 | 1.03  (0.93 to 1.14) | 0.63 | 2.27  (1.73 to 2.98) | <.0001 | 0.96  (0.77 to 1.180 | 0.67 |
| minor | 1.56  (1.39 to 1.74) | <.0001 | 1.09  (0.98 to 1.21) | 0.12 | 1.72  (1.31 to 2.26) | 0.0001 | 0.91  (0.73 to 1.130) | 0.40 |
| non-user | 0.10  (0.07 to 0.14) | <.0001 | 0.16  (0.13 to 0.21) | <.0001 | 0.21  (0.12 to 0.36) | <.0001 | 0.17  (0.10 to 0.27) | <.0001 |
| **Years since dementia diagnosis** |  |  |  |  |  |  |  |  |
| 0-1 | Reference | **-** | Reference | - | Reference | - | Reference | - |
| 2-9 | 0.99  (0.98 to 1.00) | 0.20 | 1.29  (1.26 to 1.32) | <.0001 | 1.02  (0.98 to 1.06) | 0.25 | 1.29  (1.24 to 1.34) | <.0001 |
| ≥10 | 1.01  (0.98 to 1.04) | 0.40 | 0.83  (0.79 to 0.88) | <.0001 | 1.34  (1.25 to 1.44) | <.0001 | 1.56  (1.46 to 1.67) | <.0001 |
| **# unique medications in use on index date** |  |  |  |  |  |  |  |  |
| 0-5 | Reference | **-** | Reference | - | Reference | - | Reference | - |
| 6-9 | 1.27  (1.24 to 1.30) | <.0001 |  |  |  |  |  |  |
| 10-19 | 1.30  (1.27 to 1.33) | <.0001 |  |  |  |  |  |  |
| 20+ | 1.30  (1.26 to 1.33) | <.0001 |  |  |  |  |  |  |
| **Past year hospitalization for COPD exacerbation** |  |  |  |  |  |  |  |  |
| Yes | 0.86  (0.82 to 0.89) | <.0001 | N/A |  | N/A |  | N/A |  |
| No | Reference | **-** |  |  |  |  |  |  |
| **Physician Characteristics** |  |  |  |  |  |  |  |  |
| **Physician age (years)** | 1.00  (0.99 to 1.00) | 0.01 | 1.00  (0.99 to 1.00) | 0.02 | 1.00  (1.00 to 1.01) | 0.35 | 0.99  (0.99 to 1.00) | 0.005 |
| **Physician sex** |  |  |  |  |  |  |  |  |
| Female | Reference | **-** | Reference | - | Reference | - | Reference | - |
| Male | 0.93  (0.91 to 0.95) | <.0001 | 1.05  (1.02 to 1.09) | 0.002 | 1.13  (1.07 to 1.19) | <.0001 | 1.11  (1.05 to 1.17) | 0.0003 |
| Missing data | 0.94  (0.70 to 1.26) | 0.68 | 0.84  (0.61 to 1.14) | 0.26 | 1.21  (0.79 to 1.86) | 0.38 | 1.36  (0.61 to 3.01) | 0.45 |
| **Years since medical school graduation** |  |  |  |  |  |  |  |  |
| ≤15 | Reference | - | Reference | - | Reference | - | Reference | - |
| 16-25 | 1.06  (1.02 to 1.10) | 0.004 | 1.13  (1.06 to 1.19) | <.0001 | 1.03  (0.94 to 1.13) | 0.57 | 1.07  (0.97 to 1.17) | 0.17 |
| 26-35 | 1.12  (1.06 to 1.18) | <.0001 | 1.19  (1.10 to 1.29) | <.0001 | 0.98  (0.86 to 1.11) | 0.70 | 1.19  (1.04 to 1.35) | 0.01 |
| ≥36 | 1.11  (1.03 to 1.20) | 0.004 | 1.16  (1.04 to 1.30) | 0.01 | 0.98  (0.83 to 1.17) | 0.86 | 1.27  (1.06 to 1.52) | 0.01 |
| **Canada medical graduate** |  |  |  |  |  |  |  |  |
| Yes | 1.11  (1.08 to 1.14) | <.0001 | 1.08  (1.04 to 1.12) | <.0001 | 0.91  (0.86 to 0.97) | 0.002 | 0.98  (0.93 to 1.04) | 0.51 |
| No | Reference | - | Reference | - | Reference | - | Reference | - |
| Missing data | 1.04  (0.92 to 1.18) | 0.50 | 1.10  (0.89 to 1.35) | 0.39 | 1.12  (0.78 to 1.59) | 0.55 | 0.78  (0.61 to 1.00) | 0.05 |
| **Practice location** |  |  |  |  |  |  |  |  |
| Urban | Reference | - | Reference | - | Reference | - | Reference | - |
| Non-urban | 1.02  (0.98 to 1.06) | 0.27 | 1.12  (1.07 to 1.17) | <.0001 | 1.17  (1.07 to 1.27) | 0.003 | 1.23  (1.12 to 1.36) | <.0001 |
| Rural | 0.93  (0.87 to 0.99) | 0.02 | 1.08  (1.00 to 1.17) | 0.05 | 1.19  (1.03 to 1.37) | 0.02 | 1.32  (1.16 to 1.50) | <.0001 |
| Missing data | 1.01  (0.90 to 1.14) | 0.82 | 0.95  (0.78 to1.16) | 0.63 | 0.85  (0.61 to 1.18) | 0.33 | 1.21  (0.97 to 1.50) | 0.09 |
|  |  |  |  |  |  |  |  |  |
|  |  |  |  |  |  |  |  |  |
|  |  |  |  |  |  |  |  |  |

## Table E: Complete Results of Multivariable Poisson GEE to Assess Association of Primary Care Providers’ Daily Patient Volume on Medications from ALL Prescribers Given to Older Ontarians with Dementia (n = 100, 256)

| **Model** | **Cholinesterase Inhibitor Prescription** | | **Benzodiazepine Prescription** | | **Antipsychotic Prescription** | |
| --- | --- | --- | --- | --- | --- | --- |
|  | **Adjusted RR**  **(95% CI)** | ***p*-value** | **Adjusted RR**  **(95% CI)** | ***p*-value** | **Adjusted RR**  **(95% CI)** | ***p*-value** |
| **Physician’s Daily Patient Volume** |  |  |  |  |  |  |
| <20 patients/day | Reference | - | Reference | - | Reference | - |
| 20 -24 patients/day | 1.04  (1.01 to 1.07) | 0.01 | 1.09  (1.04 to 1.14) | 0.0003 | 1.03  (0.99 to 1.07) | 0.19 |
| 25-29 patients/day | 1.06  (1.03 to 1.09) | 0.0002 | 1.13  (1.07 to 1.19) | <.0001 | 1.09  (1.04 to 1.14) | 0.0004 |
| ≥30 patients/day | 1.05  (1.02 to 1.08) | 0.001 | 1.22  (1.16 to 1.28) | <.0001 | 1.14  (1.10 to 1.19) | <.0001 |
| **Patient Characteristics** |  |  |  |  |  |  |
| **Age group** |  |  |  |  |  |  |
| Females 66-69 | Reference | - | Reference | - | Reference | - |
| Females 70-74 | 1.21  (1.14 to 1.29) | <.0001 | 0.84  (0.77 to 0.91) | <.0001 | 1.00  (0.92 to 1.08) | 0.99 |
| Females 75-79 | 1.38  (1.31 to 1.46) | <.0001 | 0.76  (0.70 to 0.82) | <.0001 | 0.93  (0.86 to 1.00) | 0.06 |
| Females 80-84 | 1.39  (1.32 to 1.47) | <.0001 | 0.69  (0.64 to 0.75) | <.0001 | 0.90  (0.83 to 0.97) | 0.01 |
| Females 85+ | 1.19  (1.13 to 1.26) | <.0001 | 0.68  (0.63 to 0.73) | <.0001 | 0.97  (0.90 to 1.04) | 0.42 |
| Males 66-69 | 0.88  (0.82 to 0.95) | 0.0013 | 0.79  (0.71 to 0.87) | <.0001 | 0.94  (0.86 to 1.04) | 0.24 |
| Males 70-74 | 1.16  (1.09 to 1.23) | <.0001 | 0.69  (0.63 to 0.75) | <.0001 | 0.90  (0.82 to 0.97) | 0.01 |
| Males 75-79 | 1.36  (1.28 to 1.44) | <.0001 | 0.57  (0.52 to 0.62) | <.0001 | 0.84  (0.78 to 0.91) | <.0001 |
| Males 80-84 | 1.37  (1.29 to 1.44) | <.0001 | 0.54  (0.50 to 0.59) | <.0001 | 0.86  (0.79 to 0.93) | <.0001 |
| Males 85+ | 1.22  (1.16 to 1.29) | <.0001 | 0.54  (0.49 to 0.58) | <.0001 | 0.88  (0.82 to 0.95) | 0.001 |
| **Neighborhood Deprivation Index** |  |  |  |  |  |  |
| 1 - least deprived | Reference | - | Reference | - | Reference | - |
| 2 | 0.98  (0.95 to 1.00) | 0.08 | 1.04  (0.99 to 1.10) | 0.12 | 1.05  (1.00 to 1.09) | 0.04 |
| 3 | 0.97  (0.95 to 1.00) | 0.03 | 1.02  (0.97 to 1.08) | 0.42 | 1.04  (0.99 to 1.08) | 0.12 |
| 4 | 0.98  (0.95 to 1.00) | 0.06 | 1.08  (1.03 to 1.14) | 0.003 | 1.04  (1.00 to 1.09) | 0.07 |
| 5 - most deprived | 0.92  (0.89 to 0.94) | <.0001 | 1.12  (1.07 to 1.18) | <.0001 | 1.07  (1.02 to 1.11) | 0.002 |
| Missing data | 0.88  (0.83 to 0.93) | <.0001 | 0.90  (0.81 to 1.004) | 0.06 | 1.01  (0.93 to 1.10) | 0.77 |
| **Immigrated to Ontario in past 10 years** |  |  |  |  |  |  |
| No | Reference | - | Reference | - | Reference | - |
| Yes | 1.003  (0.95 to 1.06) | 0.91 | 0.84  (0.74 to 0.94) | 0.003 | 0.94  (0.85 to 1.03) | 0.16 |
| **Location of dwelling** |  |  |  |  |  |  |
| urban | Reference | - | Reference | - | Reference | - |
| non-urban | 1.03  (1.00 to 1.06) | 0.05 | 1.02  (0.97 to 1.09) | 0.43 | 1.04  (0.98 to 1.10) | 0.21 |
| rural | 1.04  (0.99 to 1.10) | 0.09 | 0.98  (0.89 to 1.08) | 0.71 | 1.02  (0.94 to 1.11) | 0.66 |
| Missing data | 1.05  (0.94 to 1.19) | 0.39 | 0.96  (0.74 to 1.23) | 0.73 | 0.94  (0.77 to 1.16) | 0.60 |
| **Years since dementia diagnosis** |  |  |  |  |  |  |
| 0-1 | Reference | - | Reference | - | Reference | - |
| 2-9 | 1.14  (1.12 to 1.16) | <.0001 | 1.04  (1.01 to 1.07) | 0.01 | 1.17  (1.14 to 1.20) | <.0001 |
| ≥10 | 0.72  (0.69 to 0.75) | <.0001 | 1.27  (1.21 to 1.35) | <.0001 | 1.28  (1.22 to 1.35) | <.0001 |
| **CIHI Comorbidity Group Index (2- year look-back)** |  |  |  |  |  |  |
| no health condition | Reference | **-** | Reference | - | Reference | - |
| major and palliative | 1.10  (1.01 to 1.19) | 0.02 | 1.89  (1.53 to 2.35) | <.0001 | 1.72  (1.46 to 2.03) | <.0001 |
| moderate | 0.97  (0.89 to 1.05) | 0.43 | 1.97  (1.58 to 2.45) | <.0001 | 0.92  (0.78 to 1.09) | 0.35 |
| minor | 1.04  (0.95 to 1.13) | 0.39 | 1.50  (1.20 to 1.87) | 0.0003 | 0.95  (0.80 to 1.13) | 0.55 |
| non-user | 0.16  (0.12 to 0.20) | <.0001 | 0.17  (0.11 to 0.28) | <.0001 | 0.17  (0.11 to 0.24) | <.0001 |
| **Physician Characteristics** |  |  |  |  |  |  |
| **Physician age (years)** | 0.999  (0.997 to 1.00) | 0.33 | 1.003  (0.9995 to 1.01) | 0.08 | 1.00  (0.99 to 1.00) | 0.25 |
| **Physician sex** |  |  |  |  |  |  |
| Female | Reference | - | Reference | - | Reference | - |
| Male | 1.005  (0.98 to 1.03) | 0.69 | 1.08  (1.04 to 1.12) | 0.0002 | 1.05  (1.02 to 1.09) | 0.003 |
| Missing data | 0.87  (0.69 to 1.09) | 0.23 | 1.23  (0.86 to 1.75) | 0.25 | 1.29  (0.85 to 1.94) | 0.23 |
| **Years since medical school graduation** |  |  |  |  |  |  |
| ≤15 | Reference | - | Reference | - | Reference | - |
| 16-25 | 1.09  (1.05 to 1.13) | <.0001 | 0.97  (0.91 to 1.04) | 0.43 | 1.02  (0.96 to 1.08) | 0.48 |
| 26-35 | 1.11  (1.05 to 1.17) | 0.0002 | 0.93  (0.85 to 1.02) | 0.11 | 1.06  (0.98 to 1.14) | 0.17 |
| ≥36 | 1.10  (1.02 to 1.18) | 0.01 | 0.95  (0.83 to 1.07) | 0.39 | 1.12  (1.00 to 1.25) | 0.05 |
| **Canada medical graduate** |  |  |  |  |  |  |
| Yes | 1.02  (1.00 to 1.05) | 0.08 | 0.91  (0.88 to 0.95) | <.0001 | 0.93  (0.90 to 0.96) | <.0001 |
| No | Reference | - | Reference | - | Reference | - |
| Missing data | 1.06  (0.91 to 1.23) | 0.47 | 1.09  (0.84 to 1.42) | 0.50 | 0.81  (0.68 to 0.96) | 0.02 |
| **Practice location** |  |  |  |  |  |  |
| Urban | Reference | - | Reference | - | Reference | - |
| Non-urban | 1.01  (0.98 to 1.05) | 0.46 | 1.07  (1.005 to 1.15) | 0.04 | 1.05  (0.98 to 1.12) | 0.16 |
| Rural | 1.02  (0.97 to 1.09) | 0.42 | 1.06  (0.95 to 1.19) | 0.31 | 1.09  (1.00 to 1.20) | 0.06 |
| Missing data | 0.99  (0.85 to 1.14) | 0.85 | 0.89  (0.70 to 1.14) | 0.36 | 1.18  (1.02 to 1.38) | 0.03 |

## Table F: Interactions between Doctor-Patient Relationship Variables and High (≥30 patients/day) Daily Patient Volume in Association with Vaccination and Medication Receipt in Older Ontarians with Dementia (n = 100, 256)

| **Effects** | **Influenza Vaccination** | | **Cholinesterase Inhibitor Prescription** | | **Benzodiazepine Prescription** | | **Antipsychotic Prescription** | |
| --- | --- | --- | --- | --- | --- | --- | --- | --- |
|  | **Adjusted RR***  **(95% CI)** | ***p*-value** | **Adjusted RR**  **(95% CI)** | ***p*-value** | **Adjusted RR**  **(95% CI)** | ***p*-value** | **Adjusted RR**  **(95% CI)** | ***p*-value** |
| High volume*high frequency | 1.14  (1.09 to 1.18) | <.0001 | 1.15  (1.07 to 1.22) | <.0001 | 1.04  (0.94 to 1.16) | 0.44 | 1.06  (0.95 to 1.19) | 0.29 |
| High volume*high continuity | 1.08  (1.03 to 1.13) | 0.001 | 1.12  (1.04 to 1.21) | 0.002 | 1.09  (0.97 to 1.23) | 0.15 | 1.02  (0.91 to 1.15) | 0.73 |
| High volume*long relationship | 1.06  (1.01 to 1.12) | 0.03 | 1.03  (0.96 to 1.10) | 0.41 | 1.11  (0.996 to 1.24) | 0.06 | 1.02  (0.91 to 1.15) | 0.70 |

Notes:
*All models were adjusted for the following confounders: patient age, sex, years since dementia diagnosis, CIHI Population Grouper category, urban/rural location of residence, neighborhood deprivation quintile, past 10-year immigration, and PCP age, sex, years since graduation, Canadian medical graduate, urban/rural location of practice. Models of influenza vaccination were also adjusted for past-year hospitalization for chronic obstructive pulmonary disease.
